# Gallium-doped zinc oxide semiconductor nanoparticles for plasmonic applications: a combined experimental and computational study

**DOI:** 10.1039/d5na01093d

**Published:** 2026-01-22

**Authors:** Naga Venkateswara Rao Nulakani, Yiqiang Chen, Alessandro Genovese, Rachid Sougrat, Dalaver Hussain Anjum

**Affiliations:** a Department of Physics, Khalifa University of Science and Technology P.O. Box 127788 Abu Dhabi United Arab Emirates dalaver.anjum@ku.ac.ae; b KAUST Core Labs, King Abdullah University of Science and Technology (KAUST) Thuwal 23955 Kingdom of Saudi Arabia

## Abstract

In this study, gallium-doped zinc oxide (GZO) nanoparticles were synthesized *via* a sol–gel approach followed by controlled thermal treatment, yielding nanocrystalline semiconductors with tunable Ga concentrations for advanced plasmonic applications. Structural and morphological analyses using transmission electron microscopy (TEM) revealed uniform grain distribution with particle sizes of ∼60–80 nm and the preservation of the wurtzite crystal framework. It further confirmed the successful substitution of Zn^2+^ by Ga^3+^ ions (2.5% doping), demonstrating effective doping without disrupting the lattice integrity. The analysis of the complex dielectric function, including the real (*ε*_1_) and imaginary (*ε*_2_) components, exhibited a crossover of *ε*_1_ from negative to positive values and a corresponding peak in *ε*_2_ within the near-infrared region, indicative of strong plasmonic resonance. Complementary electron energy loss spectroscopy (EELS) revealed a sharp, intense peak near ∼0 eV, confirming the presence of collective free-carrier oscillations. To rationalize these observations, first-principles density functional theory (DFT) calculations were performed, revealing an energy gap of 3.1 eV. We have observed an upward shift of the Fermi level toward the conduction band, consistent with enhanced free-carrier density due to Ga incorporation. The emergence of partially occupied conduction states, spanning −1.3 to −1.7 eV for GZO2.5 and GZO6.25, promotes intraband transitions, leading to a pronounced low-energy optical response and a robust epsilon-near-zero (ENZ) effect. Collectively, these results highlight the coexistence of semiconducting and plasmonic behavior in GZO nanoparticles, underscoring their potential for tunable optoelectronic devices, low-loss infrared plasmonics, and ENZ-enabled photonic applications.

## Abstract 2

In this study, gallium-doped zinc oxide (GZO) nanoparticles were synthesized *via* a sol–gel approach followed by controlled thermal treatment, yielding nanocrystalline semiconductors with tunable Ga concentrations for advanced plasmonic applications.

## Graphical abstract

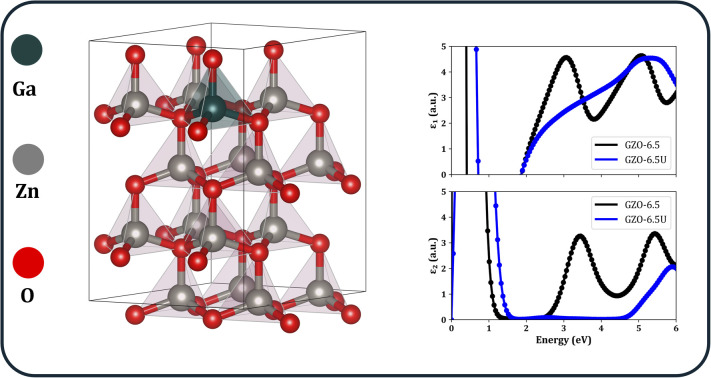

## 1 Introduction

Plasmonics^1,2^ and metamaterials^3,4^ have profoundly reshaped the landscape of nanophotonic materials by providing unprecedented control over the interaction between light and matter at the nanoscale, extending far beyond the limits imposed by classical optics.^5^ This unique capability has opened transformative pathways in subwavelength optics, leading to the development of cutting-edge technologies such as ultrasensitive biochemical sensors,^6,7^ nanoscale lasers,^8–10^ high-speed photonic interconnects,^11,12^ electromagnetic cloaking devices,^13^ imaging systems that surpass the diffraction limit, and components for quantum photonic applications.^14–17^ The central concept underlying plasmonics is the excitation of surface plasmon resonances (SPRs), which arise from the collective oscillations of conduction electrons at the interface between a metallic surface and a surrounding dielectric medium. These resonances confine and enhance electromagnetic fields within small spatial regions, thereby enabling the manipulation of light at dimensions well below its wavelength. Despite these breakthroughs, conventional plasmonic materials, including noble metals such as gold (Au) and silver (Ag), exhibit intrinsic limitations that hinder further progress.^18,19^ Although they demonstrate superior plasmonic performance in the visible and ultraviolet spectral regions, they also suffer from substantial ohmic losses, limited spectral tunability, and incompatibility with complementary metal-oxide-semiconductor (CMOS) processing technologies. These drawbacks restrict their integration into scalable photonic and optoelectronic systems. Furthermore, the inefficiency of noble metals in the near- and mid-infrared (NIR–MIR, approximately 1–10 µm) ranges significantly constrains their use in technologically important applications such as thermal imaging, infrared sensing, optical communication, and molecular fingerprint detection, where robust and tunable plasmonic responses are essential.^20^

These limitations have driven the search for alternative plasmonic materials that offer tunable optical properties, reduced losses, and better integration with existing device architectures.^18,19^ In this pursuit, transparent conducting oxides (TCOs) have emerged as a promising class of materials that combine high electrical conductivity with optical transparency.^21^ Unlike noble metals, TCOs can support tuneable plasmonic resonances in the NIR region while remaining transparent in the visible range.^22^ This makes them attractive for multifunctional plasmonic and optoelectronic applications. Among the known TCOs, indium tin oxide (ITO) has long served as the benchmark, widely used in displays, photovoltaic cells, and light-emitting diodes.^22^ However, reliance on indium, a scarce and toxic element with rising costs, introduces sustainability and scalability concerns. These issues have led to growing interest in indium-free alternatives, particularly zinc oxide (ZnO), a non-toxic, earth-abundant, and chemically stable semiconductor.^23^

ZnO exhibits a wide band gap of approximately 3.4 eV and a high exciton binding energy of around 60 meV. It also demonstrates natural n-type conductivity due to native point defects such as oxygen vacancies and zinc interstitials. However, the intrinsic carrier density of pristine ZnO is too low to support plasmonic activity in the infrared range.^24^ To overcome this, extrinsic doping is necessary. Introducing donor atoms increases the free-carrier concentration by providing electrons to the conduction band. Several dopants, including indium, tin, aluminum, silver, and gallium, have been investigated to modulate the properties of ZnO.^25–28^ Among them, gallium has emerged as a particularly effective choice. Gallium has an ionic radius similar to that of zinc, allowing it to substitute seamlessly into the ZnO lattice without introducing significant structural strain. Each gallium atom contributes an additional free electron, enhancing n-type conductivity while preserving optical transparency.^29^ Unlike aluminum, which tends to exhibit solubility limitations and clustering at higher doping levels, gallium shows stable incorporation across a wide range of concentrations. This stability allows for predictable and reproducible tuning of optical and electrical behaviour of ZnO. As a result, gallium-doped ZnO (GZO) has attracted attention as a stable, scalable, and environmentally friendly platform for plasmonic and optoelectronic technologies.

Interestingly, degenerately doped TCOs^30^ such as aluminium-doped ZnO (AZO), ITO, and GZO can exhibit negative real permittivity (*ε*_1_ < 0) in the NIR, a critical requirement for sustaining localized surface plasmon resonances.^22^ While ITO and AZO have been extensively studied for such behaviour, GZO remains relatively underexplored, particularly from a theoretical and experimental standpoint.^18^ The reliance of ITO on indium further raises concerns regarding material costs, long-term sustainability, and supply limitations. Moreover, at the high carrier concentrations required for plasmonic operation, ITO often exhibits increased damping due to reduced carrier mobility and thermal instability during post-deposition processing, thereby significantly degrading optical performance. AZO has been proposed as an indium-free alternative to ITO and has been investigated primarily for transparent electrodes. Nevertheless, aluminum doping in ZnO introduces strong ionized-impurity scattering, reducing carrier mobility at high doping levels and thereby limiting the plasmonic figure of merit. As a result, achieving low-loss, highly tunable plasmonic behavior in AZO remains challenging, particularly in the near- and mid-infrared regimes. Despite the chemical similarity between AZO and GZO, direct plasmonic comparisons between these two materials, particularly with respect to loss mechanisms, ENZ tunability, and mobility-limited damping, remain scarce in the literature. In contrast, GZO offers a favourable combination of optical performance, thermal stability, and compatibility with existing CMOS technologies, positioning it as a strong candidate for infrared plasmonic applications in communication systems, photonic circuits, and energy harvesting devices.^31–34^ In this context, GZO represents a promising yet comparatively underexplored plasmonic material. Gallium acts as an efficient donor in ZnO, potentially introducing less ionized-impurity scattering than aluminum, thereby enabling higher carrier mobility at comparable carrier concentrations.^35,36^ These characteristics suggest that GZO may offer a favorable balance between tunability, optical loss, and material stability. However, systematic investigations linking Ga doping concentration, free-carrier density, plasmonic dispersion, and loss mechanisms, particularly in direct comparison with established plasmonic oxides such as ITO and AZO, remain limited. The present work addresses this gap by providing a detailed analysis of the plasmonic response of GZO, thereby clarifying its potential as a low-loss, indium-free plasmonic material for infrared and ENZ-based photonic applications. Nevertheless, advanced experimental and computational characterization techniques are required to investigate properties of GZO across multiple length scales. For example, conventional methods such as X-ray diffraction (XRD) and scanning electron microscopy (SEM) provide valuable insights into the crystal structure and surface morphology of GZO nanoparticles.^37,38^ However, these approaches are insufficient to probe the atomic- and nanoscale features that influence plasmonic performance. This has led to growing use of advanced electron microscopy techniques, particularly transmission electron microscopy (TEM) combined with electron energy-loss spectroscopy (EELS), which have become essential for analysing doped semiconductor systems at high spatial resolution.^39,40^

Modern TEM techniques, capable of achieving sub-angstrom resolution,^41^ enable direct imaging of atomic arrangements, crystallographic defects, and dopant distributions in GZO nanoparticles.^42,43^ When used together with EELS, these techniques not only reveal structural information but also provide detailed insights into the local electronic structure and optical properties through the analysis of energy-loss spectra. EELS is particularly valuable for studying plasmonic materials, as it enables direct detection of surface and bulk plasmons, as well as interband transitions that shape the dielectric function. Using Kramers–Kronig analysis,^44^ it becomes possible to extract the complex dielectric function from EELS data, a crucial step in understanding and predicting plasmonic behaviour. Complementary techniques such as high-angle annular dark-field (HAADF) imaging and energy-dispersive X-ray spectroscopy (EDS) further contribute by enabling spatial mapping of elemental distributions, which is critical for evaluating the uniformity of dopant incorporation and identifying potential segregation.

On the other hand, theoretical modeling based on first-principles methods also plays a central role in complementing experimental observations. Density functional theory (DFT), particularly when combined with Hubbard U corrections to account for electron correlation, has been widely used to elucidate the influence of dopants on the electronic structure of semiconductors.^45^ In the case of GZO, such simulations help predict how increasing gallium concentrations drive the transition from semiconducting to metallic behaviour. These calculations provide insights into changes in electronic structure, density of states, and dielectric response, all of which are key to plasmonic functionality in the NIR region. When integrated with experimental data from TEM and EELS, these theoretical insights provide a robust framework for designing optimized doped oxide systems.

By integrating advanced microscopy, first-principles modeling, and controlled synthesis, this work establishes a comprehensive foundation for understanding and utilizing GZO in next-generation plasmonic and optoelectronic applications. The study aims to unlock the full potential of GZO as a sustainable, tunable, and low-loss alternative to noble metals in the infrared spectral region. The specific objectives of this study are as follows (i) to synthesize Ga-doped ZnO nanoparticles with controlled doping levels and characterize their structural and morphological properties using different experimental techniques, (ii) to investigate the atomic-scale structure and elemental distribution within GZO nanoparticles using advanced transmission electron microscopy (TEM) and to probe their plasmonic behaviour, and (iii) to perform density functional theory (DFT) calculations for pristine and Ga-doped ZnO at selected doping concentrations, analyzing their electronic structures, density of states (DOS), and the evolution of electronic properties that support plasmonic activity in the near-infrared region.

## 2 Methodology

### 2.1 Materials synthesis

Gallium-doped zinc oxide (GZO) nanoparticles were synthesized *via* a modified sol–gel technique to achieve precise control over composition and morphology, consistent with previous studies.^46–49^ Initially, stoichiometric amounts of zinc nitrate [Zn(NO_3_)_2_], gallium nitrate [Ga(NO_3_)_3_], and citric acid [C_6_H_8_O_7_] were carefully weighed according to the required doping levels. These reagents were dissolved in deionized water to prepare a transparent and homogeneous precursor solution. Continuous magnetic stirring was maintained for 30 minutes to promote uniform mixing and precursor coordination. The resulting solution was dried at 80 °C for at least 3 hours, yielding a xerogel. This xerogel was subsequently aged at 130 °C for 12 hours to enhance crosslinking and initiate nucleation of oxide species. The swollen and semi-rigid mass was then finely ground using an agate mortar and pestle to obtain a uniform powder. This powder was finally calcined in atmospheric air at 600 °C for 10 hours to remove residual organics and achieve crystallization of the oxide nanoparticles. Gallium doping was achieved by varying the Ga(NO_3_)_3_ concentration while maintaining a constant overall molar ratio of metal nitrates to citric acid. The synthesized nanoparticles had the general formula Ga_*x*_Zn_1−*x*_O, with *x* = 0.005 (0.5%), 0.01 (1%), and 0.025 (2.5%). For comparison, undoped ZnO nanoparticles (*x* = 0) were also synthesized under identical conditions. The precise precursor quantities used for each doping level are summarized in Table 1.

**Table 1:** Number of grams of all reagents used to synthesize Ga_*x*_Zn_1−*x*_O

Doping (%)	C_6_H_8_O_7_	Zn(NO_3_)_2_	Ga(NO_3_)_3_	Final composition
0	0.3541	0.3489	0.00000	ZnO
0.5	0.3539	0.3471	0.00236	Ga_0.005_Zn_0.995_O
1	0.3539	0.3453	0.00471	Ga_0.010_Zn_0.990_O
2.5	0.3536	0.3398	0.01176	Ga_0.025_Zn_0.975_O

### 2.2 Material characterization

Comprehensive physicochemical and optoelectronic characterization was carried out to elucidate the influence of Ga doping on the structure, morphology, composition, and optical response of the synthesized Ga doped ZnO nanoparticles. High-resolution transmission electron microscopy and electron energy loss spectroscopy were performed on a Titan Themis Z transmission electron microscope (Thermo Fisher Scientific) operated at 80 kV. The instrument is equipped with a monochromator and double aberration correction, providing an energy resolution of approximately 40–45 meV, which is essential for resolving low-energy excitations in the near-zero-loss regime. The microscope is integrated with a Gatan Imaging Filter (GIF) Continuum spectrometer and advanced direct electron detectors, including K3 and STELLA, thereby enabling enhanced signal sensitivity and a high signal-to-noise ratio for subtle energy-loss features. Beam damage was minimized by selecting the low accelerating voltage of 80 kV, which is below the knock-on displacement threshold for Zn and O atoms in ZnO, while still providing sufficient signal quality for low-loss EELS. Low probe currents and short dwell times in microprobe STEM mode further reduced the total electron dose, limiting radiolysis and local heating effects. High-sensitivity direct electron detectors allowed acquisition under these low-dose conditions without prolonged exposure. Spectra were collected from fresh, uniform regions of individual nanoparticles, and repeated irradiation of the same area was avoided. Continuous monitoring of the zero-loss peak showed no broadening or intensity loss, confirming negligible beam-induced modification of the sample.

The electron beam was monochromatized and operated in microprobe STEM mode to balance spatial and energy resolution. Microprobe STEM mode was deliberately selected over conventional parallel-beam TEM-EELS modality to achieve an optimal balance among spatial resolution, momentum resolution, and signal stability for nanoscale plasmonic measurements. In this configuration, the convergence semi-angle is maintained at a low value while preserving a localized probe, resulting in a quasi-parallel beam with reduced momentum spread (Δ*k*).^50^ Such conditions favor dipole-dominated scattering and ensure the validity of the dielectric approximation required for reliable low-loss plasmon analysis and subsequent Kramers–Kronig evaluation. In addition, microprobe STEM enables localized probing of individual nanoparticles, thereby minimizing contributions from surrounding regions, substrate effects, and thickness variations that can obscure weak low-energy plasmonic features in parallel beam TEM mode. Before performing Kramers–Kronig analysis (KKA), particular care was taken to ensure that the acquired low-loss EELS spectra were dominated by bulk plasmon excitations rather than surface-related modes. Spectra were collected from sufficiently thick, uniform interior regions of individual nanoparticles, where volume plasmon contributions prevail, and surface plasmon excitation probabilities are significantly reduced. During acquisition, microprobe STEM mode was employed with a small convergence semi-angle and a controlled collection angle—these conditions preferentially sample low-momentum-transfer scattering events associated with dipole-allowed bulk excitations. The probe's spatial position was carefully controlled *via* simultaneous STEM imaging to avoid nanoparticle edges and surfaces, where surface plasmon modes are strongest. As a result, the low-loss spectra consistently exhibited a single dominant plasmon peak, without additional low-energy shoulders or multiple resonances indicative of mixed surface and bulk contributions. Thickness effects were explicitly considered by estimating the relative sample thickness (*t*/*λ*) directly from low-loss EELS spectra using the log-ratio method, where *t* is the specimen thickness and *λ* is the inelastic mean free path. For the regions selected for KKA, *t*/*λ* values ranged from approximately 0.2 to 0.4, corresponding to thicknesses of approximately 15–30 nm, depending on local density and composition. This regime minimizes plural scattering, and spectra were acquired from uniform interior regions with careful zero-loss peak subtraction. The absence of spectral broadening, artificial peak shifts, or unphysical behavior in *ε*_1_ and *ε*_2_ confirms that thickness-related artifacts were negligible, and results were consistent across multiple particles and acquisition locations. The processed spectra were then subjected to Kramers–Kronig analysis (KKA) under the dielectric approximation, facilitating the extraction of the real and imaginary parts of the complex dielectric function, denoted as *ε*_1_(*ω*) and *ε*_2_(*ω*), respectively.^44^ The reliability of the extracted dielectric functions was ensured by combining high energy resolution, a low convergence semi-angle, careful ZLP subtraction, and acquisition from controlled interior regions. Internal consistency checks confirmed that zero crossings of *ε*_1_ coincided with peaks in *ε*_2_ and maxima in the energy loss function, as expected for genuine plasmonic excitations. Additionally, trends in *ε*_1_ and *ε*_2_ were in good qualitative agreement with independently calculated dielectric responses from density functional theory, particularly for the enhanced low-energy intraband contributions introduced by Ga doping, confirming that the extracted dielectric response faithfully reflects the intrinsic plasmonic behavior.

This comprehensive characterization protocol enabled rigorous correlation among particle size, dopant distribution, and optical excitations. It provided an in-depth understanding of how Ga doping modifies the dielectric environment and influences the plasmonic properties of GZO nanoparticles.

### 2.3 Computational methods

First-principles calculations were performed using both spin-polarized and non-spin-polarized density functional theory (DFT) as implemented in the Vienna *Ab Initio* Simulation Package (VASP).^51–53^ The interaction between valence and core electrons was treated accurately using the projector augmented wave (PAW) formalism proposed by Blöchl.^54^ For the exchange-correlation functional, the generalized gradient approximation (GGA) in the Perdew–Burke–Ernzerhof (PBE) scheme was adopted, given its proven reliability in describing the equilibrium geometries, bonding characteristics, and electronic structures of oxide and doped semiconductor systems.^55–58^ A plane-wave energy cutoff of 520 eV was employed to guarantee stable total energies and precise forces on each atom. The ionic positions and lattice constants were fully relaxed until the Hellmann–Feynman forces on each atom became smaller than 0.001 eV Å^−1^, and the total energy difference between successive electronic steps was less than 10^−8^ eV, ensuring structural and electronic self-consistency. Brillouin zone integrations were carried out using Monkhorst–Pack *k*-point meshes with reciprocal-space resolutions finer than 0.02 Å^−1^ for structural optimization and 0.01 Å^−1^ for electronic structure calculations.^59^ All computations were performed under periodic boundary conditions to simulate bulk-like behavior and capture the intrinsic electronic characteristics of both pristine and Ga-doped ZnO models.

All optical properties of a material can be derived from its complex dielectric function, *ε*(*ω*), which characterizes the response of the material to an external electromagnetic field as a function of frequency (*ω*) or wavelength. The dielectric function consists of two parts:*ε*(*ω*) = *ε*_1_(*ω*) + *iε*_2_(*ω*)where *ε*_1_(*ω*) is the real part, describing dispersion, and *ε*_2_(*ω*) is the imaginary part, representing absorption. The following expressions give these components:
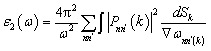

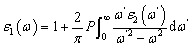
Here, *P*_*nn*′_(*k*) represents the dipole transition matrix elements, *ω*_*nn*′_(*k*) is the energy difference between the initial and final states, *dS*_*k*_ denotes integration over the Brillouin zone surface, and *P* indicates the Cauchy principal value of the integral.

## 3 Results and discussion

### 3.1 Experimental results

#### 3.1.1 Structural and morphological analysis

**Fig. 1:**
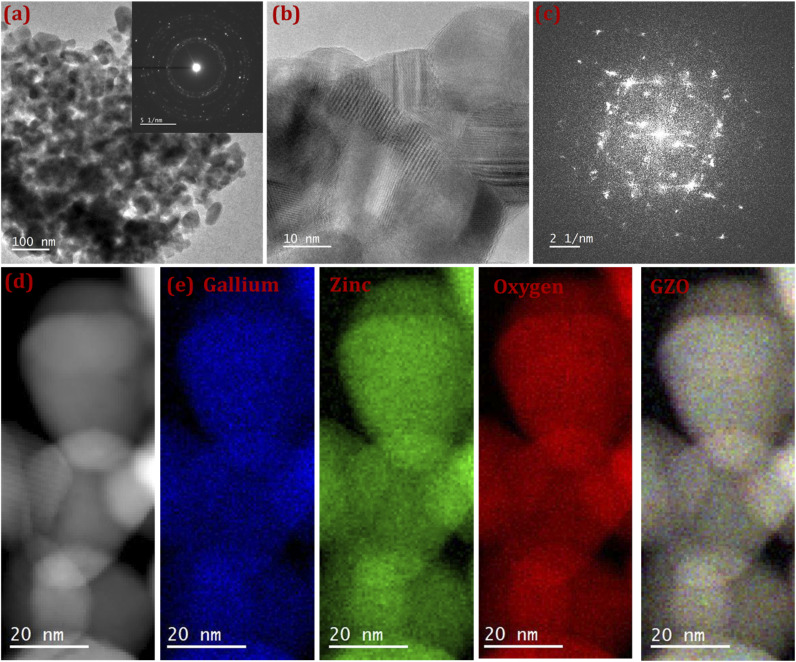
TEM analysis of 2.5% Ga-doped ZnO nanoparticles. (a) Low-magnification TEM with SAED inset confirming crystallinity, (b) HRTEM showing lattice fringes, (c) FFT pattern verifying structural order, (d) ADF-STEM image, and (e) EDS-generated elemental maps of Ga, Zn, and O showing uniform Ga distribution across the ZnO matrix.

Fig. 1 presents a detailed transmission electron microscopy (TEM) investigation of Ga-doped ZnO (GZO) nanoparticles synthesized at a gallium doping concentration of 2.5%, aimed at characterizing their morphology, crystallinity, and elemental distribution. In Fig. 1a, the low-magnification TEM image reveals a dense agglomeration of nanoparticles with nearly spherical to slightly faceted morphologies. The nanoparticles are relatively monodisperse, with sizes ranging from approximately 60 to 80 nm, indicating a narrow size distribution achieved under optimized synthesis conditions. The inset of Fig. 1a displays a selected area electron diffraction (SAED) pattern composed of a series of concentric diffraction rings, which are characteristic of polycrystalline materials. The sharp, well-defined nature of these rings suggests that the GZO nanoparticles are highly crystalline and that multiple crystallites contribute to the diffraction pattern.^60^ The observed ring spacings are consistent with the wurtzite-type ZnO crystal structure, and no additional rings corresponding to Ga-related secondary phases (*e.g.*, Ga_2_O_3_) are observed, confirming the phase purity of the synthesized material. Consistent with these observations, the XRD patterns of such highly doped GZO samples exhibit diffraction peaks that can be exclusively indexed to the hexagonal wurtzite structure of ZnO, with no additional reflections attributable to Ga_2_O_3_ or other impurity phases within the detection limit of the technique, thereby confirming phase purity at the bulk scale. Fig. 1b presents the high-resolution TEM (HRTEM) image of an individual nanoparticle, clearly resolving lattice fringes across several domains. These fringes correspond to distinct crystallographic planes, and the measured interplanar spacings (*d*-spacings) are in close agreement with the (100), (002), and (101) planes of hexagonal wurtzite ZnO, suggesting that Ga substitution occurs without disrupting the long-range periodicity of the ZnO lattice. The crystallographic coherence and sharp lattice contrast throughout the nanoparticle provide further evidence of its single-crystalline or twinned polycrystalline nature. The inset in Fig. 1c shows the fast Fourier transform (FFT) of the HRTEM image, exhibiting bright, discrete diffraction spots arranged in hexagonal symmetry. This confirms the well-ordered atomic arrangement and corroborates the high crystallinity inferred from both the SAED and HRTEM observations. No additional spots or diffuse scattering features are observed in the FFT pattern, indicating structural integrity and the absence of amorphous phases or significant lattice distortion attributable to gallium incorporation. In Fig. 1d, an annular dark-field (ADF) image is shown, which provides Z-contrast-based visualization of the nanoparticle region. The image enhances the contrast between elements of differing atomic number, making it particularly suitable for elemental mapping using energy-dispersive X-ray spectroscopy (EDS). Fig. 1e provides EDS elemental maps corresponding to gallium (blue), zinc (green), and oxygen (red), each obtained from the same particle ensemble shown in the ADF image. The Ga signal, although relatively weak due to its low doping concentration, is consistently distributed across all nanoparticle regions, indicating homogeneous incorporation of Ga into the ZnO host matrix. The Zn and O maps show strong and uniform intensity, consistent with the stoichiometry of the ZnO framework. The final composite image labeled “GZO” combines all three elemental maps, indicating that Ga is not only present but also well dispersed within each nanoparticle. Importantly, there are no signs of Ga clustering or segregation at grain boundaries or surfaces, confirming that the doping process has led to successful substitutional or interstitial incorporation of Ga ions rather than to the formation of separate Ga-rich domains. Overall, the microstructural analysis strongly supports the successful synthesis of phase-pure, highly crystalline, and uniformly doped Ga : ZnO nanoparticles at a Ga content of 2.5%.

The optical bandgap energies of the synthesized samples were determined comprehensively in our previous study.^60^ For the undoped ZnO nanoparticles, the extracted bandgap was approximately 3.25 eV, slightly lower than the commonly reported value for bulk ZnO (3.37 eV). Upon gallium doping at 2.5%, the bandgap decreased marginally to 3.23 eV. This subtle reduction in bandgap energy upon Ga incorporation is consistent with prior observations and can be attributed to the introduction of structural and chemical disorder in the material. Specifically, Ga doping can lead to a high density of intrinsic defects, such as oxygen vacancies or zinc interstitials, particularly in intergranular regions where atomic coordination is less uniform. These defects introduce localized electronic states within the band structure, effectively narrowing the bandgap by facilitating sub-band transitions. Additionally, the slight red shift in the absorption edge seen in the doped sample supports this interpretation. Therefore, the observed reduction in optical bandgap in Ga-doped ZnO not only reflects the changes in electronic structure induced by dopant incorporation but also provides insight into the underlying defect chemistry and its influence on the optical properties of the material.

#### 3.1.2 Optical properties

**Fig. 2:**
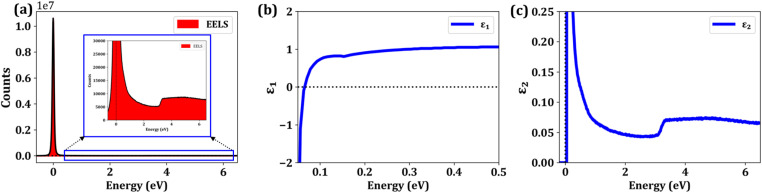
Optoelectronic response of 2.5% Ga-doped ZnO (GZO2.5). (a) electron energy-loss spectroscopy (EELS) spectrum Panel (c) presents the corresponding, highlighting the low-energy plasmonic features. The complex dielectric function is decomposed into its (b) real (*ε*_1_) and (c) imaginary (*ε*_2_) components.

The optical response of Ga-doped ZnO (GZO) at a doping concentration of 2.5% was thoroughly investigated by evaluating the real and imaginary components of the complex dielectric function, along with the electron energy loss spectrum (EELS) are presented in Fig. 2. The energy loss function, defined as the imaginary component of the inverse dielectric function Im[−1/*ε*(*ω*)], was computed and plotted in Fig. 2a. A sharp, intense plasmon resonance is observed in the low-energy region near the zero-energy limit, characterized by high intensity and a narrow linewidth. Such a spectral feature is a clear indication of low damping and reduced plasmonic losses, both essential characteristics of high-quality plasmonic materials.

This behaviour is consistent with previous theoretical and experimental investigations of transparent conducting oxides, particularly AZO, in which heavy doping leads to the formation of a free-electron gas and a pronounced low-energy plasmon mode. In AZO systems, the emergence of a strong EELS peak has been attributed to collective oscillations of conduction electrons introduced by dopant atoms, with the plasmon frequency being susceptible to carrier concentration and effective mass. Similar to AZO, the strong plasmonic response observed in GZO confirms that Ga substitution efficiently increases the electron density without inducing substantial structural disorder or excessive scattering centers, thereby preserving low optical losses.

Further insight into the collective excitation phenomena is obtained from the real part of the dielectric function, *ε*_1_(*ω*), depicted in Fig. 2b. In the low-energy regime below approximately 0.1 eV, *ε*_1_(*ω*) becomes negative, indicating metallic optical behavior dominated by free carriers. As photon energy increases, *ε*_1_ crosses zero and then becomes positive, marking the system's screened plasma frequency. This negative-to-positive crossover is a hallmark of plasmonic activity in doped semiconductors and defines the transition between reflective and transmissive optical regimes. Comparable behavior has been extensively reported for AZO, where *ε*_1_ diverges negatively as *ω* approaches zero, in agreement with Drude-like free-electron behavior and experimental infrared ellipsometry measurements. The agreement between GZO and AZO in this regard confirms that Ga doping induces a similar metallic response in ZnO, validating the applicability of the Drude–Lorentz framework for describing the low-energy optical response.

The imaginary component of the dielectric function, *ε*_2_(*ω*), shown in Fig. 2c, further supports this interpretation. A pronounced low-energy peak is observed, corresponding to strong intraband transitions that give rise to free-carrier absorption in the infrared region. This feature mirrors the rapid decay of *ε*_2_ reported for AZO at low frequencies, which is attributed to viscous electron damping arising from electron-phonon and impurity scattering processes that are linked to electrical resistivity. As the photon energy increases, *ε*_2_ decreases and remains relatively flat across the visible spectrum, ensuring high optical transparency, a defining characteristic of transparent conducting oxides. A modest rise in *ε*_2_ near approximately 3.23 eV is attributed to the onset of interband transitions, consistent with the fundamental absorption edge of ZnO-based materials. Further, the optical band gap values of the pristine and Ga-doped ZnO samples were also determined using the Tauc plot method in our previous work. The Tauc plots were constructed assuming a direct allowed transition, consistent with the well-established direct band gap nature of ZnO.^61,62^ For undoped ZnO, the extracted band gap was approximately 3.25 eV, while for Ga-doped ZnO (*x* = 0.025) a slightly reduced band gap of ∼3.23 eV was obtained. The slight reduction in band gap upon Ga doping is attributed to the combined effects of dopant-induced disorder, defect-related localized states, and band tailing near the conduction band edge.

Notably, similar to AZO, the interband absorption edge in GZO remains well separated from the low-energy plasmonic regime. In AZO, Al doping induces a hypsochromic shift of the absorption edge due to the Burstein–Moss effect, while simultaneously shifting the plasma frequency from the far-ultraviolet region of pristine ZnO toward the near-infrared and visible ranges. Analogously, the present GZO system exhibits a plasmon resonance confined to the low-energy infrared regime without overlapping the interband transition energies above approximately 3.3 eV. This separation is critical because it minimizes optical losses in the infrared and visible regions, thereby addressing a key limitation of conventional noble metals. Indeed, experimental studies on AZO films have demonstrated optical losses up to five times lower than those of silver in the near-infrared region, highlighting the technological relevance of doped ZnO systems.

Overall, the combined dielectric and EELS analyses demonstrate that GZO at 2.5 percent doping exhibits a highly tunable optical response with strong low-energy plasmonic character and low intrinsic losses. The close correspondence between the optical behavior of GZO and well-studied AZO systems confirms that Ga doping is equally effective in transforming ZnO into a plasmonically active transparent conducting oxide. These findings underscore the suitability of GZO for infrared plasmonics, low-energy photonic devices, and plasmon-enhanced optoelectronic applications, where controllable plasma frequency, reduced damping, and strong light-matter interaction are of paramount importance.

### 3.2 Density functional theory calculations of ZnO

The experimental findings prompted a theoretical investigation to uncover the mechanistic details of plasmonic behaviour in GZO. To this end, a detailed atomistic study was conducted using density functional theory (DFT). These calculations elucidate the precise cause-and-effect relationship between Ga doping and emergent optoelectronic properties, mapping the evolution of the electronic structure and optical constants of ZnO as a function of dopant concentration at the most fundamental level.

#### 3.2.1 Structural properties

**Fig. 3:**
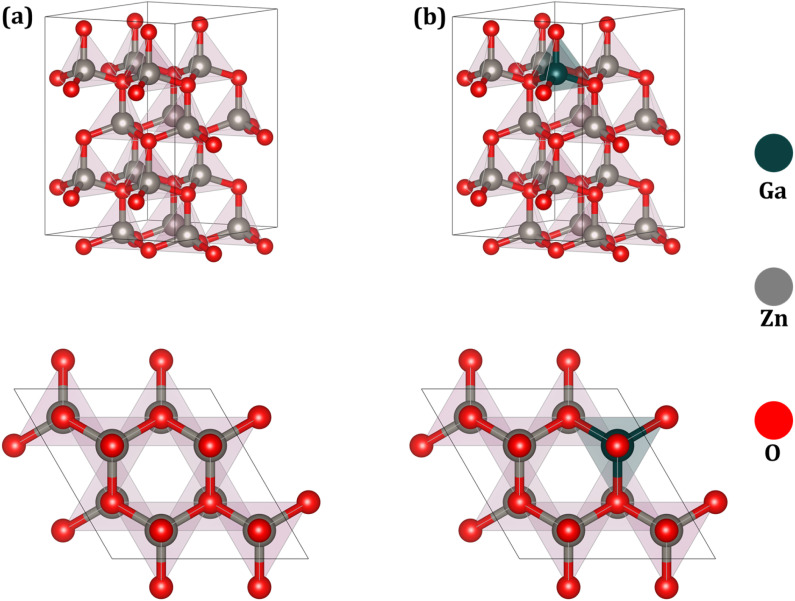
Atomic structure of the 2 × 2 × 2 supercell for (a) pristine ZnO and (b) Ga-doped ZnO (GZO) at a concentration of 6.25%. The doped structure illustrates the substitution of a single Zn atom (gray) with Ga (blue).

Fig. 3a depicts the fully optimized hexagonal wurtzite supercell of pristine ZnO, comprising sixteen zinc (Zn) and sixteen oxygen (O) atoms. The optimized lattice parameters of the ZnO unit cell are determined to be *a* = *b* = 3.287 Å and *c* = 5.307 Å, consistent with previous theoretical and experimental reports. Fig. 3b illustrates the structural configurations of Ga-doped ZnO at a 6.25% doping concentration. The Ga-doped structures were constructed by substituting a single Zn atom with Ga in 2 × 2 × 2 and 5 × 2 × 2 ZnO supercells, corresponding to 6.25% and 2.5% Ga doping, respectively. The Ga-doped ZnO models were labeled with doping percentages to simplify the discussion. For example, 6.25% Ga-doped ZnO is indicated as GZO6.25, and 2.5% Ga-doped ZnO is indicated as GZO2.5. The optimized lattice parameters of pristine ZnO are *a* = 3.287 Å and *b* = 5.307 Å, while for GZO2.5, *a* = 3.290 Å and *b* = 5.313 Å, and for GZO6.25, *a* = 3.292 Å and *b* = 5.327 Å per one cell. It indicates that the lattice constants and the volume of GZO2.5 and GZO6.25 are slightly increased due to the Ga doping when compared to those of pure ZnO. Furthermore, the increment is proportional to the doping percentage. The pristine ZnO exhibits the three in-plane Zn–O bonds of a length of ∼2.004 Å, while it is around 2.011 Å in the out-of-plane Zn–O bond. These values are slightly increased in GZO2.5 and GZO6.25. For example, the in-plane Zn–O bonds are increased up to 2.067 Å (2.070 Å), while the out-of-plane Zn–O bond increased up to 2.082 Å (2.046 Å) in GZO6.25 (GZO2.5). In both cases, the Ga–O bond is comparatively shorter than those of in-plane (1.918) and out-of-plane (1.910) Zn–O bonds. These slight modifications occurred in the Zn–O bond lengths due to the presence of Ga leads to further changes in the overall lattice constants.

#### 3.2.2 Electronic properties of Ga-doped ZnO

**Fig. 4:**
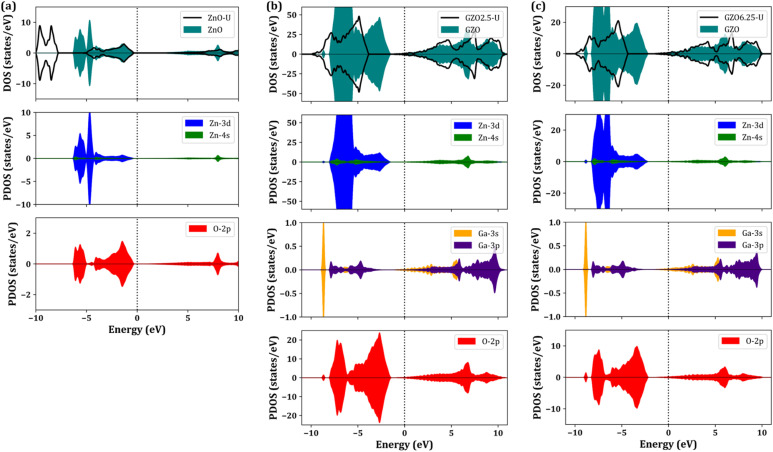
Projected and total density of states (PDOS/TDOS) for (a) pristine ZnO, (b) GZO2.5, and (c) GZO6.25. Calculations performed with standard DFT are compared directly with those incorporating a Hubbard U correction (DFT + U).

This section presents a comprehensive analysis of the electronic structure of pure and Ga-doped ZnO, highlighting their characteristic electronic fingerprints. The partial and total density of states (DOS) for pristine ZnO, GZO2.5, and GZO6.25 are shown in Fig. 4. Our calculations reveal that pristine ZnO behaves as a semiconductor with a narrow direct band gap of 0.72 eV at the PBE-GGA level of theory, consistent with previously reported theoretical studies (see Fig. S1). It is well recognized that the PBE-GGA functional systematically underestimates the band gap of semiconductors due to its intrinsic limitations in treating electron–electron interactions, particularly in localized d states. In the case of ZnO, the PBE-GGA predicted gap of 0.72 eV is considerably smaller than the experimentally determined value of 3.4 eV. To address this discrepancy, we employed the Hubbard U correction for the Zn d states (denoted as ZnO–U) to better account for on-site Coulomb interactions. The resulting total DOS, as illustrated in Fig. 4a, indicates an expanded band gap of approximately 3.2 eV, which aligns closely with experimental observations and previous theoretical reports, validating the reliability of the U-corrected approach (see Fig. S1). The analysis of the TDOS and PDOS further elucidates the orbital contributions near the Fermi level. The valence band is primarily composed of O 2p states, hybridized with Zn 3d orbitals, which dominate the electronic states just below the Fermi level. This p-d hybridization is critical in determining the electronic and optical properties of ZnO. The conduction band, on the other hand, is primarily characterized by Zn 4s states, with a contribution from O 2p orbitals, indicating that the low-lying conduction states are mainly associated with Zn s and O 2p-like character. This orbital decomposition provides a clear picture of the electronic transitions responsible for optical absorption and offers a foundation for understanding the modifications introduced by Ga doping.

Doping ZnO with Ga induces substantial modifications in the electronic structure, as reflected in both the TDOS and PDOS of the doped models. Fig. 4b and c present the TDOS and PDOS of GZO2.5 and GZO6.25, respectively, clearly illustrating the doping-induced electronic changes. In the case of GZO2.5 (see Fig. S2), the Fermi level shifts noticeably towards the conduction band, indicating that Ga acts as an electron donor, effectively resulting in n-type doping. This behaviour arises from the extra valence electron in group III elements, such as Ga^3+^, relative to Zn^2+^, which introduces additional electrons into the conduction band and imparts metallic character to the system. The observed semiconductor-to-metal transition in GZO2.5 is particularly significant, highlighting its potential for plasmonic and metamaterial applications, where tunable free-carrier concentrations are essential. For GZO6.25, with a higher Ga concentration, the Fermi level shift becomes even more pronounced (see Fig. S3), reflecting the accumulation of additional conduction electrons and the enhancement of metallic behaviour. Analysis of the DOS for both GZO2.5 and GZO6.25 confirms that Ga doping does not generate prominent mid-gap defect states within the ZnO band gap. Instead, the primary effect of Ga incorporation is to promote the Fermi level into the unoccupied region, thereby acting as a donor of charge carriers. This controlled doping behaviour ensures that the electronic structure remains intact, primarily by facilitating carrier injection. The absence of deep-level defect states can be attributed to the relatively low Ga concentration, which induces only minor perturbations to the atomic structure of the pristine ZnO lattice. Consequently, the Ga dopants enhance conductivity without compromising the fundamental semiconducting framework, making GZO2.5 and GZO6.25 excellent candidates for low-loss, tunable plasmonic and optoelectronic devices.

It is noteworthy that the valence states of ZnO near the Fermi level remain largely unaffected by the inclusion of the Hubbard U parameter, indicating that the occupied states are relatively insensitive to on-site Coulomb interactions. In contrast, the conduction band minimum shifts significantly upward, leading to an overall increase in the band gap (see Fig. 4a and S1). This behaviour arises from the enhanced on-site repulsion between the Zn-3d and O-2p orbitals, which pushes the unoccupied conduction states to higher energies while leaving the occupied valence states essentially unchanged. In ZnO with the Hubbard correction (ZnO-U), the upper valence band is primarily composed of hybridized Zn-3d and O-2p orbitals, whereas Zn-4s states predominantly characterize the conduction band with minor contributions from O-2p orbitals. The valence states exhibit a broader distribution in the energy range of 0 to −5.5 eV and are more localized around −7.5 eV, reflecting the complex interplay between delocalized and localized electronic states. A similar electronic pattern is observed in Ga-doped ZnO systems under the influence of U. In these doped models, such as GZO2.5-U and GZO6.25-U, the Fermi level moved to the unoccupied conduction region, intersecting the DOS due to the additional valence electrons introduced by Ga. This electron excess induces n-type conductive behaviour, consistent with experimental observations for Ga-doped ZnO. As with ZnO-U, the inclusion of the Hubbard potential increases the separation between the conduction and valence bands.

Two dominant trends characterize the evolution of the electronic structure with Ga doping. First, the Fermi level is progressively shifted to higher energies, penetrating deeper into the conduction band. Second, and equally important, is a pronounced broadening of the energy window of occupied conduction states. In GZO2.5, the occupied conduction states span approximately 1.3 eV before the DOS reaches a minimum near −1.3 eV, reflecting the marginal distribution of electronic states around the Fermi level. For GZO6.25, this occupancy range extends to roughly 1.7 eV, with the DOS reaching a minimum near −1.7 eV. This systematic broadening illustrates the pronounced influence of Ga on the electronic structure, enhancing carrier density and metallic character in a controlled manner. The progressive evolution of DOS with increasing doping concentration provides critical insight into the correlation between Fermi level shifts and the resulting optical and transport properties. Such tunable electronic behaviour is fundamental to engineering Ga-doped ZnO for use as transparent conducting oxides, low-loss plasmonic materials, and near-infrared optoelectronic devices, where precise control over carrier concentration is essential.

#### 3.2.3 Dielectric properties of Ga-doped ZnO

**Fig. 5:**
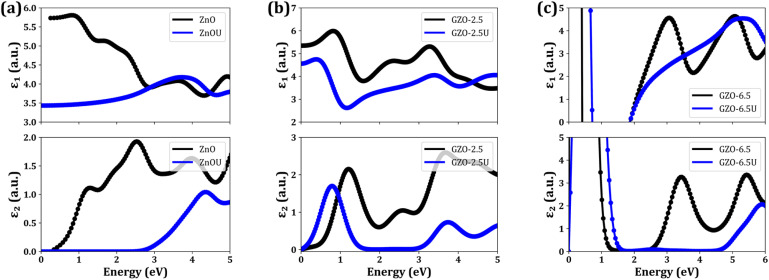
Calculated complex dielectric functions for (a) pristine ZnO, (b) GZO2.5, and (c) GZO6.5. The real (*ε*_1_) and imaginary (*ε*_2_) components are plotted, comparing the results obtained with and without the inclusion of a Hubbard U correction.

In this section, we present a detailed exploration of the optical properties of both pure and Ga-doped ZnO, focusing on the results derived from the partial density of states (PDOS) and total density of states (TDOS) discussed in the previous section. Specifically, we have calculated the real component, *ε*_1_(*ω*), and the imaginary component, *ε*_2_(*ω*), of the complex dielectric response for pure ZnO, as well as for the Ga-doped ZnO samples, including the doping concentrations of 2.5% (GZO2.5) and 6.25% (GZO6.25). The computed results are illustrated in Fig. 5. The absorption edge of pristine ZnO, as predicted by the PBE-GGA functional, occurs at roughly 0.7 eV, exhibiting a substantial underestimation relative to experimental data (see Fig. 5a). This deviation is characteristic of standard semi-local functionals, which fail to capture the strong electronic correlations present in ZnO, leading to an inaccurate band gap. To address this, the application of a Hubbard U correction to the optical spectrum calculations leads to a blue shift in the absorption edge, resulting in a significantly improved representation of the optoelectronic structure of the of pure ZnO (*i.e.*, ZnOU). The corrected optical spectrum, incorporating the U parameter, closely aligns with experimental data, yielding an optical band gap of approximately 3.1 eV. The dielectric response, as observed in these calculations, is primarily driven by interband transitions, which are indicative of the semiconducting nature of ZnO.

However, upon doping Ga into ZnO to form GZO2.5, we observed the emergence of a new peak at low energy, a feature absent in the optical spectrum of undoped ZnO as shown in Fig. 5b. This feature can be attributed to electronic transitions from partially occupied Ga-3s donor states near the Fermi level to Zn-4s and Zn-4p acceptor states near the conduction band. This suggests that Ga doping introduces additional donor levels near the Fermi level, thereby facilitating low-energy optical response by enabling electronic transitions not observed in undoped ZnO. Interestingly, as in pure ZnO, the application of the Hubbard U correction (GZO2.5-U) shifts the absorption edge slightly to the blue. This shift can be attributed to the stabilization of the valence bands in GZO2.5-U. As the valence bands are stabilized, the energy required to excite electrons into the conduction band increases, resulting in a higher excitation threshold for interband transitions. Consequently, the interband transitions in GZO2.5-U occur at marginally higher energies compared to GZO2.5, reflecting the subtle but noticeable influence of Ga doping and electron correlation on the electronic and optical properties of ZnO.

Raising the Ga concentration to 6.25% results in a progressive blue shift of the absorption edge (GZO6.25-U), as shown in Fig. 5c. This is because higher Ga doping stabilizes the valence bands relative to those of GZO2.5U, thereby requiring higher excitation energy for interband transitions. Further, the optical response of GZO6.25 reveals striking features in both the imaginary (*ε*_2_) and real (*ε*_1_) components of the complex dielectric function, which are directly linked to its electronic structure. The imaginary part *ε*_2_ exhibits an extremely high, nearly divergent response in the near-zero-energy region, behavior that is well explained by the Drude model and arises from free-carrier intraband transitions. This pronounced low-energy response in the optical spectrum provides direct evidence for the accumulation of substantial electronic density near the Fermi level. This abundance of readily accessible electronic states is a prerequisite for the efficient low-energy electronic excitations. As the incident photon energy is increased, a monotonic decrease in the imaginary component of the dielectric function (*ε*_2_) is observed, culminating in a minimum around 1.7 eV. This trend mirrors the reduction in the accumulated electronic density below the Fermi level, which leads to a diminished rate of electronic transitions from initial (occupied) to final(unoccupied) states. At higher excitation energies, the electronic structure becomes more complex. The DOS undergoes a precipitous increase within a specific energy window, approximately between −3 eV and −1.5 eV relative to Fermi level. This sharp rise in the density of states manifests distinctly in the optical properties, producing a broad plateau in the *ε*_2_ spectrum. A well-defined absorption edge marks the high-energy flank of this plateau at approximately 2.5 eV. This edge signifies the energetic threshold for a new, dominant channel of interband transitions, originating from this region of high DOS in the valence band and terminating in available conduction-band states. In addition, the real component of the dielectric function, *ε*_1_, exhibits a strikingly complementary profile. It crosses the zero-axis at approximately 0.8 eV (∼1.5 µm), a wavelength in the telecommunication-relevant near-infrared (NIR) regime. At this epsilon-near-zero (ENZ) point, *ε*_1_ approaches zero, leading to strong light confinement and minimal energy dissipation. The material thus undergoes a transition from dielectric to metallic behavior, a property that can be exploited for advanced photonic and plasmonic applications. In the ENZ regime, GZO6.25 exhibits a small but negative *ε*_1_, enabling its use in the design of epsilon-near-zero and negative-refractive-index metamaterials that operate in the NIR. Remarkably, the imaginary part of *ε* in this spectral region remains several times smaller than that of conventional low-loss metals such as silver, highlighting the superior optical performance of GZO6.25. This combination of tunable permittivity, low intrinsic losses, and metallic behavior in the NIR positions GZO6.25 as an auspicious material for low-loss plasmonic devices, negative-refractive-index metamaterials, and integrated photonic systems. Its optical characteristics, which stem directly from the electronic density of states and interband transition dynamics, offer precise control over light-matter interactions. Consequently, GZO6.25 holds potential for next-generation optoelectronic and photonic technologies where low-loss, tunable, and ENZ-enabled functionalities are crucial.

**Fig. 6:**
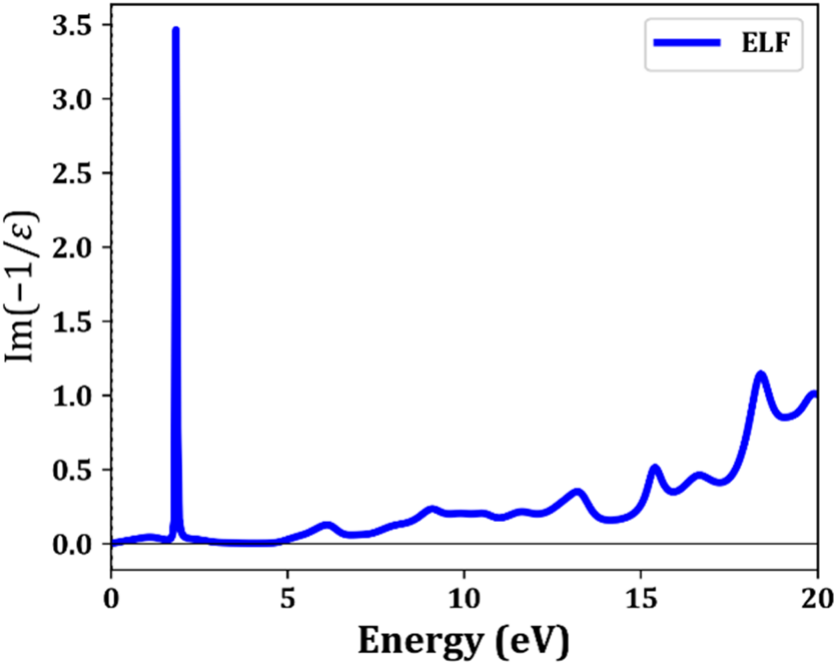
Calculated electron energy loss function (ELS) of GZO6.25 with U.

To gain a deeper understanding of the plasmonic properties of Ga-doped ZnO, we computed the electron energy loss function (ELF), Im(−1/*ε*), which directly probes the energy dissipation of fast-moving electrons. This function is closely linked to plasmon excitations, with ELF peaks corresponding to collective oscillations of free charge carriers. Fig. 6 displays the ELF spectrum for GZO6.25, incorporating Hubbard U corrections to capture electron correlation effects accurately. A sharp peak near 2.0 eV, indicative of bulk plasmon resonance, emerges due to the collective motion of electrons introduced by Ga substitution. This peak arises under resonance conditions in which the real part of the dielectric function approaches zero, and the imaginary part remains sufficiently low, thereby enabling coherent plasmon oscillations with minimal damping. Substituting Ga^3+^ for Zn^2+^ increases the free carrier density, enhancing the metallic character of the dielectric response and amplifying plasmonic activity at low energies. This results in a plasmon resonance near 2.0 eV, placing GZO6.25 in a favorable regime for low-energy plasmonic applications, particularly in the near-infrared (NIR) range. Compared with noble metals, the lower carrier density of GZO6.25 reduces the plasmon frequency, thereby enabling the design of tunable plasmonic devices at relevant wavelengths. The combination of tunable plasmon resonance and low intrinsic losses in transparent conducting oxides makes Ga-doped ZnO ideal for NIR plasmonic circuits, sensing platforms, and low-loss metamaterials.

Collectively, these observations position GZO as a highly promising plasmonic semiconductor that bridges the gap between traditional metals and semiconductors. Its combination of metallic-like optical behavior and intrinsically lower energy losses enables the realization of devices with strong light-matter coupling and improved efficiency. Such features make GZO particularly attractive for next-generation plasmon-enhanced photovoltaics, optical and chemical sensors, and engineered metamaterials where precise control over plasmon resonance is crucial. Moreover, the tunability of plasmonic response *via* doping strategies provides a versatile platform for developing NIR-active photonic and optoelectronic components, highlighting GZO6.25 as a cornerstone material for advanced low-loss plasmonic and metamaterial applications.

## 4 Conclusions

In this study, Ga-doped ZnO (GZO) nanoparticles were synthesized *via* a sol–gel method to investigate their potential for plasmonic applications. TEM analysis confirmed uniform nanoparticles with sizes ranging from ∼60–80 nm and the successful substitution of Zn^2+^ by Ga^3+^ ions at different doping concentrations (2.5% and 6.25%), demonstrating effective incorporation without disrupting the wurtzite lattice. Selected area electron diffraction (SAED) patterns, in combination with X-ray diffraction (XRD) analysis, confirmed the absence of secondary phases, indicating phase-pure, highly crystalline nanoparticles. Systematic changes in crystallinity, morphology, and electronic structure were observed with increasing Ga concentration. First-principles density functional theory calculations with the Hubbard U correction (DFT + U) supported these experimental observations and provided detailed insight into the atomic-level effects of Ga incorporation. The substitution of Zn^2+^ by Ga^3+^ introduces additional electrons into the conduction states, which span to −1.3 eV and to −1.7 eV for GZO2.5 and GZO6.25, respectively. It increases the electron concentration, metallizing the electronic structure and creating partially filled states that enable intra-band electronic transitions. This is reflected in an increased optical response, particularly an infinite contribution to the imaginary component (*ε*_2_) of the complex dielectric function near the epsilon-near-zero (ENZ) region, indicating strong plasmonic resonance. Together, the experimental and theoretical findings demonstrate that GZO exhibits tunable, low-loss plasmonic behaviour in the near-infrared region. This makes GZO a promising material for photonic and metamaterial applications, with potential to advance plasmonic technologies and optical devices.

## Author contributions

Naga Venkateswara Rao Nulakani: writing – review & editing, writing – original draft, visualization, validation, methodology, investigation, formal analysis, data curation. Yiqiang Chen: methodology, investigation, formal analysis, data curation. Alessandro Genovese: writing – review & editing, Rachid Sougrat: writing – review & editing. Dalaver Hussain Anjum: writing – review & editing, writing – original draft, visualization, validation, supervision, software, resources, project administration, methodology, investigation, funding acquisition, formal analysis, data curation, conceptualization.

## Conflicts of interest

There are no conflicts to declare.

## Supplementary Material

NA-OLF-D5NA01093D-s001

## Data Availability

Data will be made available on request. Supplementary information (SI): computational results of the electronic properties of gallium doped zinc oxide. See DOI: https://doi.org/10.1039/d5na01093d.
