# Supplementary material for: Gallium-doped zinc oxide semiconductor nanoparticles for plasmonic applications: a combined experimental and computational study
Source: Nanoscale Adv. 2026 Jan 22;8(5):1530–42. doi: 10.1039/d5na01093d (PMC12834044; doi:10.1039/d5na01093d)
Supplement: NA-008-D5NA01093D-s001 [file NA-008-D5NA01093D-s001.pdf]

Supplementary Information (SI) for Nanoscale Advances.
This journal is © The Royal Society of Chemistry 2026

Supporting Information for

Gallium-Doped Zinc Oxide Semiconductor Nanoparticles for Plasmonic Applications: A Combined Experimental and Computational Study

Naga Venkateswara Rao Nulakani,[a] Yiqiang Chen,[b] Alessandro Genovese,[b] Rachid Sougrat,[b] and Dalaver Hussain Anjum*[a]

[a.] Department of Physics, Khalifa University of Science and Technology, P.O. Box 127788, Abu Dhabi, UAE.

[b.] KAUST Core Labs, King Abdullah University of Science and Technology (KAUST), Thuwal 23955, Kingdom of Saudi Arabia.

*Corresponding Author
Email: dalaver.anjum@ku.ac.ae (**Dalaver Hussain Anjum**)

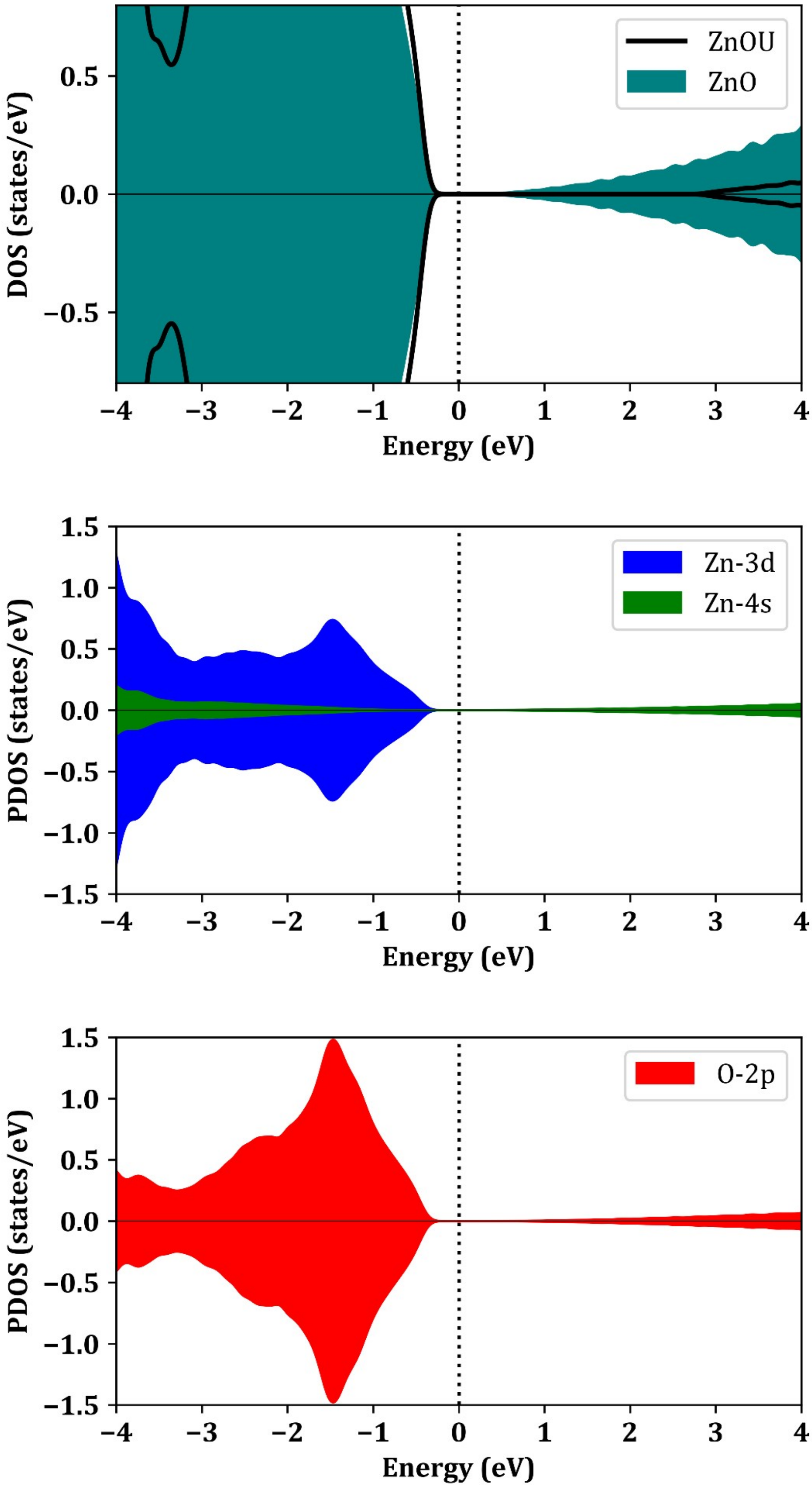

Figure S1. Projected and total density of states (PDOS/TDOS) for pristine ZnO

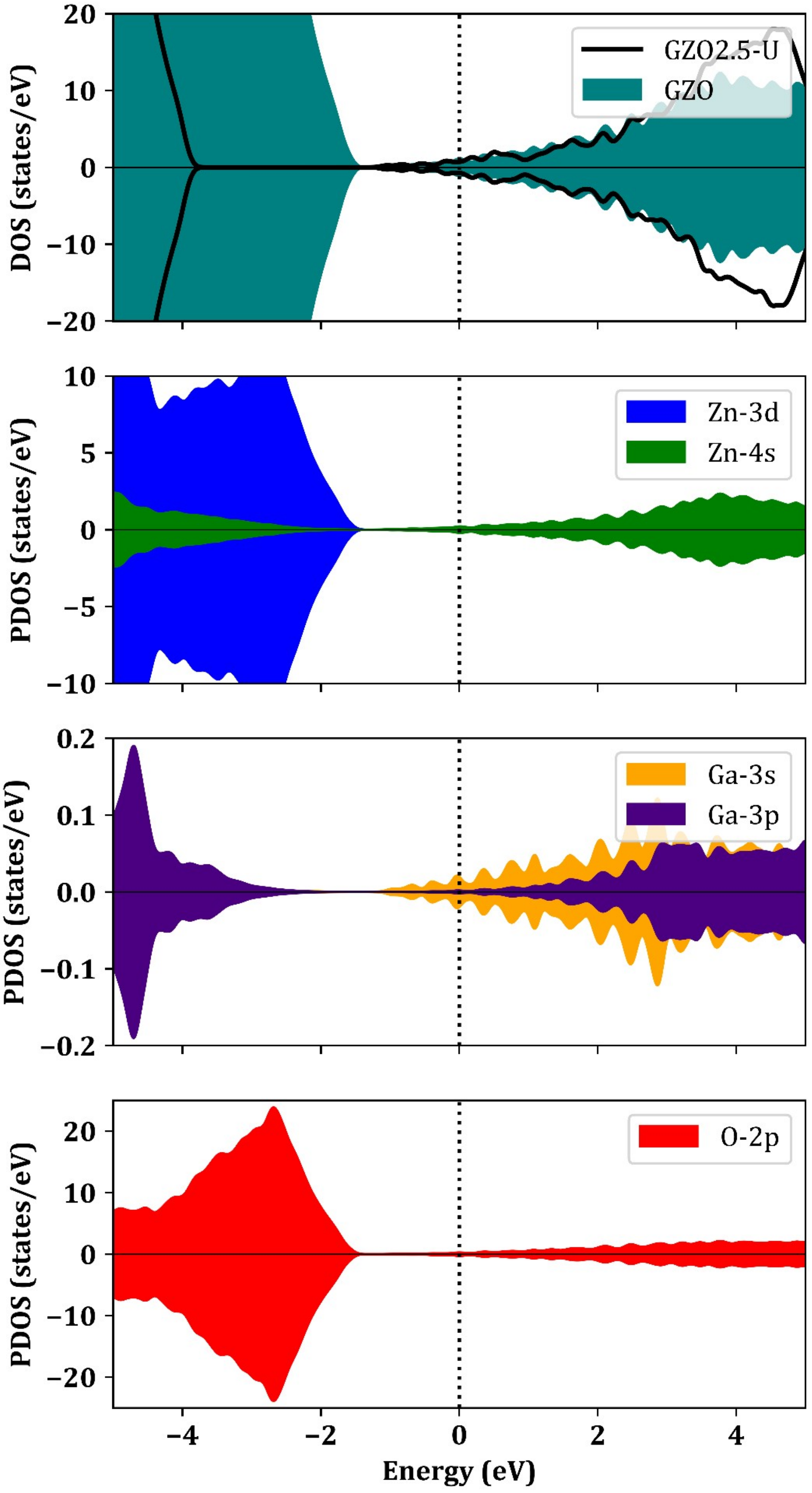

Figure S2. Projected and total density of states (PDOS/TDOS) for GZO2.5

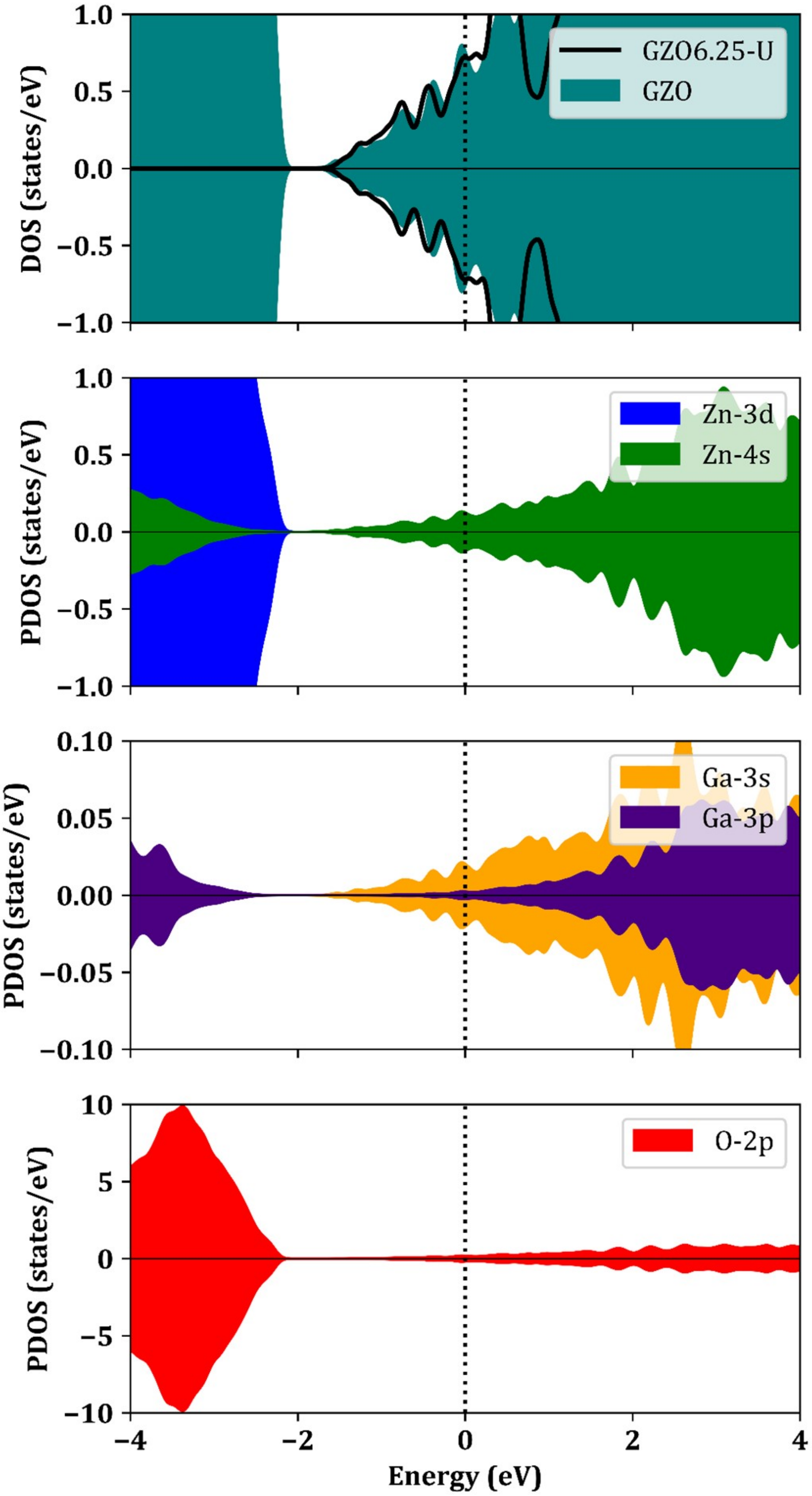

Figure S3. Projected and total density of states (PDOS/TDOS) for GZO6.25.
